# Pre-heating mitigates composite degradation

**DOI:** 10.1590/1678-775720150284

**Published:** 2015

**Authors:** Jessika Calixto da SILVA, REGES Rogério Vieira, Inara Carneiro Costa REGE, Carlos Alberto dos Santos CRUZ, Luís Geraldo VAZ, Carlos ESTRELA, Fabrício Luscino Alves de CASTRO

**Affiliations:** 1 - Consultório particular, Goiânia, GO, Brasil.; 2 - Universidade Paulista, Faculdade de Odontologia, Instituto de Ciências da Saúde, Materiais Dentários e Dentística, Goiânia, GO, Brasil.; 3 - Universidade Paulista, Faculdade de Odontologia, Instituto de Ciências da Saúde, Radiologia Oral, Goiânia, GO, Brasil.; 4 - Universidade Estadual Paulista, Faculdade de Odontologia, Departamento de Materiais Dentários e Prótese, Araraquara, SP, Brasil.; 5 - Universidade Federal de Goiás, Faculdade de Odontologia, Departamento de Ciências Estomatológicas, Goiânia, GO, Brasil.; 6 - Faculdade União de Goyazes, Curso de Odontologia, Dentística, Trindade, GO, Brasil.

**Keywords:** Composite resins, Hot temperature, Radiography, Silver nitrate, Scanning electron microscopy

## Abstract

Dental composites cured at high temperatures show improved properties and higher degrees of conversion; however, there is no information available about the effect of pre-heating on material degradation.

**Objectives:**

This study evaluated the effect of pre-heating on the degradation of composites, based on the analysis of radiopacity and silver penetration using scanning electron microscopy/energy-dispersive X-ray spectroscopy (SEM/EDS).

**Material and Methods:**

Thirty specimens were fabricated using a metallic matrix (2x8 mm) and the composites Durafill VS (Heraeus Kulzer), Z-250 (3M/ESPE), and Z-350 (3M/ESPE), cured at 25°C (no pre-heating) or 60°C (pre-heating). Specimens were stored sequentially in the following solutions: 1) water for 7 days (60°C), plus 0.1 N sodium hydroxide (NaOH) for 14 days (60°C); 2) 50% silver nitrate (AgNO_3_) for 10 days (60°C). Specimens were radiographed at baseline and after each storage time, and the images were evaluated in gray scale. After the storage protocol, samples were analyzed using SEM/EDS to check the depth of silver penetration. Radiopacity and silver penetration data were analyzed using ANOVA and Tukey’s tests (α=5%).

**Results:**

Radiopacity levels were as follows: Durafill VS<Z-350<Z-250 (p<0.05). The depth of silver penetration into the composites ranked as follows: Durafill VS>Z-350>Z-250 (p<0.05). After storage in water/NaOH, pre-heated specimens presented higher radiopacity values than non-pre-heated specimens (p<0.05). There was a lower penetration of silver in pre-heated specimens (p<0.05).

**Conclusions:**

Pre-heating at 60°C mitigated the degradation of composites based on analysis of radiopacity and silver penetration depth.

## INTRODUCTION

The interaction between composite resins and the moist oral environment can negatively affect the properties of the material. When composites are immersed in water, two different mechanisms occur: first, water sorption produces mass increase and softening of the polymer matrix; second, solubility causes components in the bulk of the material to leach to the external environment^10^ ^,^ ^27^. With time, the water inside the composite may break chemical bonds of a single polymer chain, between two or more polymer chains, and/or between silane and fillers, reducing mechanical properties and thus decreasing the durability of the material^10^.

Several factors can affect the sorption and solubility of composites, e.g., resin matrix composition, size and distribution of filler particles, and energy parameters used during polymerization^10^ ^,^ ^13^ ^,^ ^14^ ^,^ ^27^. Regarding the material composition, composites are commonly classified according to the average size and distribution of filler particles in the resin matrix; composites with different filler contents show distinct sorption and solubility behaviors^13^ ^,^ ^18^. Materials with higher filler contents tend to be more resistant to water sorption, as the amount of organic matrix available to absorb water is lower. Conversely, swelling and matrix plasticization have been observed around filler particles, as well as a reduction in tensile strength and hardness, behaviors attributed to the degradation of bonds between organic and inorganic matrices^28^.

Studies have sought ways to improve both short- and long-term behaviors of composites by testing different polymerization parameters. Pre-heating, for instance, has been shown to result in higher degrees of conversion when the composite is cured at high temperatures, probably due to the enhanced molecular mobility and greater number of collisions of reactive species achieved during high-temperature polymerization^2^ ^,^ ^5^ ^,^ ^6^ ^,^ ^8^ ^,^ ^23^. It is likely that this higher degree of conversion causes the free volume within the polymer network to reduce, which may be responsible for the lower sorption and solubility found in pre-heated composites compared to materials cured at room temperature^3^. Thus, hypothetically, the higher degree of conversion produced by pre-heating could cause these composites to degrade less. Another advantage is the lower viscosity reached with high temperatures, which has been shown to improve marginal adaptation and decrease microleakage^11^ ^,^ ^15^.

The silver nitrate staining technique has been used to investigate degradation of composites^1^ ^,^ ^16^. Water softens the composite by penetrating the matrix, leaching out unreacted monomers and fillers, which allows silver to penetrate. In addition, radiopacity has been shown to hypothetically predict composite degradation, as best-cured composites have been found to be more radiopaque^17^.

Despite the fact that pre-heating may improve the long-term behavior of composites, to date, no study has been conducted to evaluate the degradation of pre-heated composites, especially comparing different materials.

The aim of this study was to evaluate the effect of pre-heating on the degradation of different commercial composites - Durafill VS (Heraeus Kulzer, São Paulo, SP, Brazil), Z-250 (3M/ESPE, Sumaré, SP, Brazil), and Z-350 (3M/ESPE, Sumaré, SP, Brazil), by analyzing radiopacity and silver penetration using scanning electron microscopy/energy-dispersive X-ray spectroscopy (SEM/EDS). The null hypothesis was that neither the temperature nor the type of composite would influence material degradation.

## MATERIAL AND METHODS

The methods here employed to induce specimen degradation using NaOH, and to evaluate silver penetration by SEM/EDS were based on a previous study^1^.

**Figure 1:**
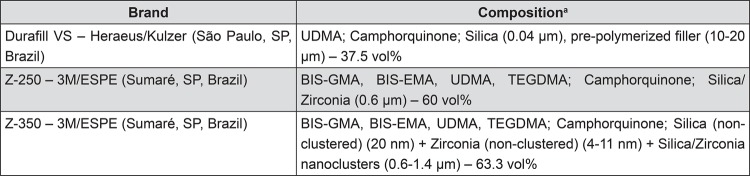
Composition of the assessed composite resins

^a^BIS-GMA: bisphenol-A glycidyl dimethacrylate; BIS-EMA: ethoxylated bisphenol-A dimethacrylate; UDMA: urethane dimethacrylate; TEGDMA: triethylene glycol dimethacrylate

Thirty specimens (n=5) were fabricated using three commercial composite resins that were cured at temperatures of 25°C (no pre-heating) and 60°C (pre-heating). Specimens were prepared with the aid of an 8x2 mm circular metallic matrix. The composition of the materials is described in detail in Figure 1.

Pre-heated specimens (cured at 60°C) were fabricated using a non-commercial heater^3^. The metallic matrix was positioned on the heater, the composite was inserted in the matrix in a single increment using a Centrix syringe, and then covered with a polyester strip and a glass coverslip. At this point, composite temperature was measured using an infrared thermometer with an accuracy of ±1°C (G-Tech, Model IR1DB1, Accumed Medical-Hospital Products Ltda., Duque de Caxias, RJ, Brazil) by touching the glass cover slip. Heater temperature was also controlled using a stick thermometer, again with an accuracy of ±1°C (Model MV-363, Minipa Indústria e Comércio Ltda., São Paulo, SP, Brazil). The two devices (metallic stick and infrared sensor) have different heat acquisition mechanisms and were used one after the other to control composite temperature. Both devices confirmed the expected temperature for the conditions evaluated in all measurements.

Once the desired temperature was reached (60°C with the heater on, or 25°C with the heater off), the resin was cured for 40 s using a LED curing light device (Radii Cal, Southern Dental Industries, Bayswater, Victoria, Australia). Light intensity was measured before the polymerization of each specimen by a radiometer coupled to the curing device to ensure a power density of >600 mW/cm^2^. Room temperature and humidity were controlled using a digital thermometer with an accuracy of ±1°C (Model MT -241, ETL- Electronics Tomorrow Ltda., China, imported by Minipa Indústria e Comércio Ltda., São Paulo, SP, Brazil).

Specimens were radiographed using intraoral X-ray equipment (Spectro 70-X, Seletronic, Dabi Atlante, Ribeirão Preto, SP, Brazil) on a phosphor plate sensor with an exposure time of 0.3 s and a focus-film distance of 40 cm. Images were digitally treated (Cliniview Dental Imaging Software 10.0.2, Instrumentarium Dental, Tuusula, Finland), exported, and then analyzed using Adobe Photoshop CS6 (Adobe Systems Inc., San Jose, California, USA). Analysis was performed using the elliptical marquee and the histogram tools, in grayscale, at a resolution from 0 to 255 pixels; five predefined 20x20-pixel areas, one in the center and the others at the periphery of the specimens, were selected for analysis, and mean radiopacity values were calculated.

Specimens were then stored in amber glass vials containing 1.5 mL of distilled water for 7 days at 60°C in an incubator (Model TE-3941, TECNAL-Equipment for Laboratories, Piracicaba, SP, Brazil). Subsequently, they were removed from the vials, dried with absorbent paper and stored again in a 0.1 N sodium hydroxide (NaOH) solution (pH 12) for 14 days, at 60°C. At this point, specimens were removed from the vials, washed in running water for 1 min, dried with absorbent paper, radiographed, and analyzed again as previously described.

Finally, specimens were stored in a 50% silver nitrate (AgNO_3_) aqueous solution, at 60°C in an incubator, for 10 days. After this time, specimens were washed in running water for 5 min, immersed in developing solution (Eastman Kodak Company, Rochester, NY, USA) and exposed to fluorescent light for 8 h. Then, specimens were washed in running water for 3 min, dried with absorbent paper, and radiographed. Radiographs were evaluated again as previously described.

Subsequently, each specimen was sectioned into three parts using a diamond disc mounted on a low-speed handpiece, and each part was embedded in composite resin (Natural Flow, Nova DFL, Rio de Janeiro, RJ, Brazil), with the cut surface exposed. Surfaces were wet-ground sequentially using silicon carbide papers (600-grit, 1200-grit, 2000-grit, 2400-grit, and 4000-grit). Then, they were polished using 3 and 0.75 μ grit diamond pastes and felt discs (Erios Technical and Scientific Equipment Ltda., São Paulo, SP, Brazil), cleaned under ultrasonic vibration for 10 min, dried, and kept in an environment containing silica gel.

For scanning electron microscopy, samples were coated with a 250 A-thick layer of gold film (Desk V, Denton Vacuum LLC, Moorestown, NJ, USA) and analyzed in a JSM-6610 microscope (JEOL Inc., Peabody, MA, USA) equipped with an EDS device (NSS ThermoScientific Spectral Imaging, Thermo Fisher Scientific Inc., Waltham, MA, USA). The depth of silver penetration was measured by quantitative digital linear scanning of 50 spots across each specimen’s surface at an accelerating voltage of 15 kV, and a magnification of 300x. A total of 15 measurements were obtained for each specimen (five measurements for each of the three specimen parts), and a mean value was calculated. Additionally, electron photomicrographs of the surfaces were taken in backscatter mode, at an accelerating voltage of 12 kV and 1000x, 2700x, and 10000x magnifications.

Two-way ANOVA was used to analyze data considering two fixed criteria, namely type of composite and curing temperature, for both radiopacity and silver penetration depth. *Post hoc* comparisons of different composites were performed using Tukey’s test. Radiopacity results associated with different storage media were compared using ANOVA and Tukey’s test for paired data. Significance was set at α=5%. Statistical analysis was performed using the Statistical Package for the Social Sciences (SPSS) (IBM SPSS Statistics 22, IBM Corp., Armonk, NY, USA).

## RESULTS

### Radiopacity

**Figure 2:**
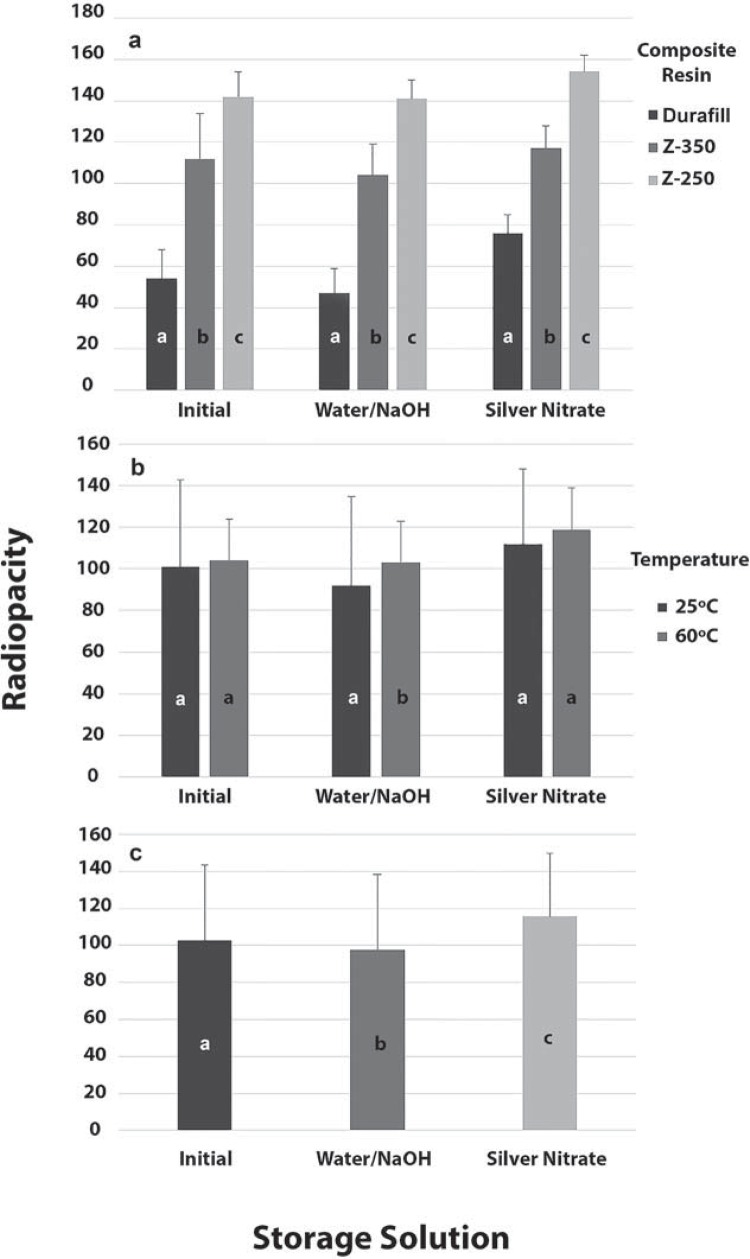
Mean radiopacity values obtained for the three composite resins assessed a) according to storage solution (Tukey’s test); b) according to curing temperature and storage solution (ANOVA); and c) according to storage solution only (Tukey’s test for paired data). Different letters indicate statistically significant differences (p<0.05)

The ANOVA test showed a statistically significant effect of type of composite on the variables of interest regardless of storage medium (p<0.001). Analysis with the Tukey test revealed the following ranking of radiopacity: Durafill VS<Z-350<Z-250 (p<0.05). Composites cured at 60°C were more radiopaque than the ones cured at 25°C after immersion in water/NaOH medium (p<0.05), but no differences were found between the two temperatures at the first evaluation or after storage in AgNO_3_ (p>0.05). Also, no significant differences were found for the interaction between the two factors (p>0.05). Radiopacity results obtained considering the different storage conditions assessed were as follows: AgNO_3_>initial>water/NaOH (p<0.05). These results are illustrated in Figure 2.

### Silver penetration

**Figure 3:**
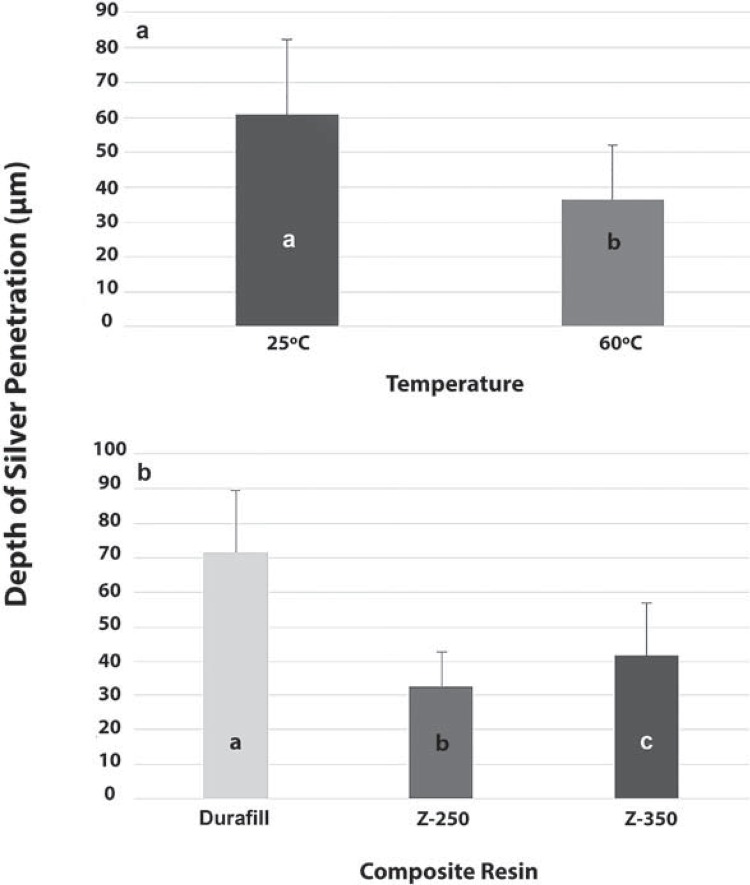
Mean values obtained for depth of silver penetration: a) according to temperature (ANOVA); and b) according to type of composite resin (Tukey’s test). Different letters indicate statistically significant differences (p<0.05)

**Figure 4:**
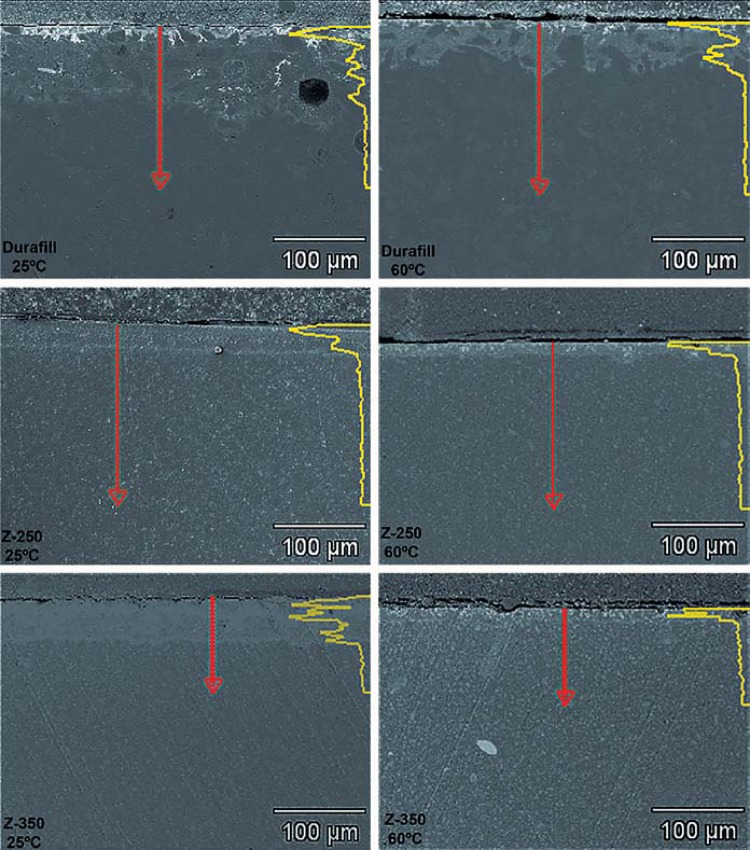
Patterns of silver penetration obtained via linear analysis with EDS, in association with different temperatures and composites. The length of the waved portion of the yellow line determines the depth of silver penetration. Red arrows indicate the direction of the quantitative digital linear scanning across each specimen’s surface

The ANOVA test revealed a significant effect of both temperature and type of composite on silver penetration depth (p<0.05). However, no statistical significance was found for the interaction between the two variables (p>0.05). Silver penetration was lower in the composites cured at 60°C than in those cured at 25°C (p<0.05). *Post hoc* comparisons showed the following ranking of silver penetration results: Durafill VS>Z-350>Z-250 (p<0.05) (Figure 3). Figure 4 shows the patterns of silver penetration resulting from the different experimental conditions investigated.

**Figure 5:**
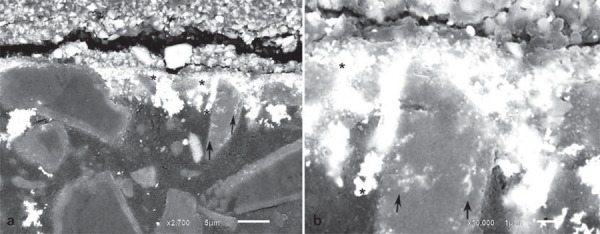
Backscattered electron micrographs of Durafill VS resin impregnated with silver. a) Extensive silver penetration over the first µm of the surface (x2,700). Note penetration of silver particles around fillers (*) and inside pre-polymerized filler particles (arrows). b) Higher magnification of the same area (x10,000)

**Figure 6:**
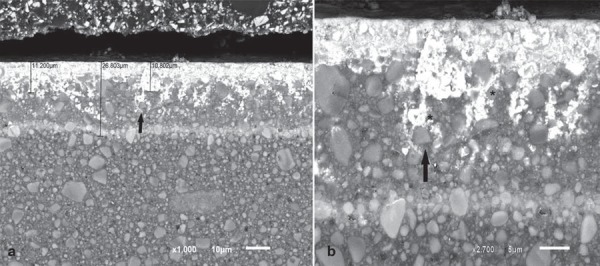
Backscattered electron micrographs of Z-250 resin impregnated with silver. a) Extensive silver penetration over approximately 11 µm of the surface, reaching 26.803 μm (x1,000). The arrow shows silver surrounding a filler particle. b) Higher magnification of the same area (x10,000). The arrow shows silver surrounding a filler particle; the asterisk (*) shows no evidence of silver within the particle

**Figure 7:**
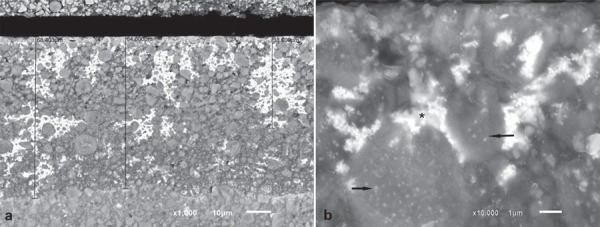
Backscattered electron micrographs of Z-350 resin impregnated with silver. a) Extensive silver penetration over approximately 68.40 µm into the subsurface. The metal is concentrated at specific sites (x1000). b) Another specimen of the same resin group impregnated with silver. The asterisk (*) shows metal surrounding nanoclustered fillers; the arrow shows metal inside nanoclusters (x10000)

Analysis of the electron micrographs revealed extensive silver penetration into the first microns below the surface of the composite, with attenuation of this penetration thereafter. Silver was also observed around filler particles. Figures 5, 6, and 7 show silver-impregnated composites.

## DISCUSSION

In our study, pre-heated composites presented higher radiopacity and a lower depth of silver penetration in comparison to composites cured at room temperature, suggesting lower degradation of the former. Even though the results of the present investigation may have been affected by composite formulation, the effects of pre-heating on both radiopacity and silver penetration were observed for all materials, regardless of the type of composite used.

Some studies had already pointed to a direct relation between pre-heating and the degree of conversion of composites^2^ ^,^ ^5^ ^,^ ^6^ ^,^ ^8^ ^,^ ^23^. High temperatures increase monomer mobility, collisions among molecules, and the amount of molecular bonds formed, improving both the degree of conversion and crosslinking – and consequently leading the polymer matrix to absorb less solvent and lose less components to the external environment, and degrade more slowly. The small space between polymer chains and the decreased amount of hydrophilic sites within the polymer would lead to these effects^10^ ^,^ ^14^ ^,^ ^27^. Lower degrees of sorption and solubility have been reported in the literature for resins heated at 60°C with specific combinations of curing times and temperatures^3^. The results of the present investigation confirm those findings. Since the phenomenon investigated in our study was limited to the surface/subsurface of the specimens, further investigation, e.g., with mechanical testing, would be necessary to confirm the association between pre-heating and degradation.

Regarding radiopacity, two phenomena may explain the results observed. First, the intermolecular distance between monomers is approximately 0.3 to 0.4 nm before polymerization, due to the nature of the bonding (Van der Waals forces). That distance decreases to approximately 0.15 nm after polymerization, by the formation of covalent bonds^20^. This denser molecular arrangement could explain the increased radiopacity of pre-heated composites^17^. Second, another aspect that could explain the increased radiopacity of pre-heated composites is the lower solubility of the material in these conditions, which probably causes fillers to leach less to the external environment. The water absorbed inward diffuses through the resin pores and other defects inside the matrix, and then slowly expels filler particles containing heavy metals – the ones responsible for composite radiopacity^10^ ^,^ ^12^.

The resin composites evaluated in the present study showed the following ascending order of radiopacity: Durafill VS<Filtek Z-350<Filtek Z-250 (p<0.05). This result was expected, as the Durafill VS resin has only silica (no zirconia) in its composition (atomic number [Z] of silica=14), and shows a lower amount of filler when compared with the other materials assessed (Figure 1). The Z-250 composite, in turn, has both silica and zirconia (Z of zirconia=40) in its composition, which increases radiopacity (Figure 1). Finally, the composition of Filtek Z-350 composite is similar to that of Z-250, but probably with a higher silica/zirconia ratio, which may explain the intermediate radiopacity values found for this material.

Silver penetration was lower in our pre-heated composites, probably because of the lower sorption and solubility associated with the pre-heating process (best-cured composites). NaOH solutions degrade the polymer network, allowing unreacted monomers and fillers to leach out from the polymer subsurface, thus creating spaces to which both water and silver nitrate are driven^1^. Taking into consideration that silver nitrate acts like a marker for water penetration^16^, we may speculate that silver penetration depth is directly associated with degradation.

*In vitro* degradation of composites using NaOH solutions has been proposed in recent years as an alternative to traditional phosphate buffer use. A literature review on the topic has found that, when accelerated degradation is required, solutions with a very high or a very low pH should be used^22^. According to another study, greatest rates of degradation are achieved with alkaline media^1^. Yet, another investigation found that alkaline media (rather than acidic media or water alone) are more suitable for the degradation of dental composites^4^. Our results support those findings, as the lowest degree of radiopacity in our specimens was obtained after storage in water/NaOH (compared with both the initial assessment and the results obtained after storage in AgNO_3_; p<0.05). The mechanism of alkaline degradation probably consists of the interaction between hydroxyl ions and the ester bonds of resinous monomers. Alkaline hydrolysis of esters in aqueous media and at high temperatures (saponification) occurs by the action of the OH-ion, a nucleophilic reagent that attacks the electrophilic bond of carbonic ester groups, replacing OR’ with OHR’ groups^29^. Therefore, the lower degradation observed for pre-heated composites probably occurred due to the higher degree of conversion and higher crosslink density associated with pre-heating, which hampered the action of NaOH on existing ester bonds in dimethacrylate monomers.

Water sorption depends on the presence of free volume within the polymer, on the affinity between polymer groups and water, and on the polymer’s resistance to deformation due to swelling^25^. Dimethacrylate-based polymers are highly dense glassy polymers. The crosslinks formed between polymer chains often cause a significant reduction in polymer permeability to solvents because they reduce the total free volume and the polymer chains’ ability to swell^25^. Comparing the results obtained for silver penetration in the different composites after immersion in AgNO_3_ aqueous solution, higher values were found for Durafill VS, followed by Z-350 and finally Z-250. The total free volume present in the polymer determines the diffusion of solvents inside it and depends on molecular packing. Flexible polymer chains with polar groups, especially those forming hydrogen bonds that increase intermolecular attractions, favor packing^25^.

The free volume present in polymers depends on their structure. Homopolymer sorption has been classified as follows: poly-TEGDMA (6.33%)>poly-Bis-GMA (2.93%)>poly-UDMA (2.59%)>poly-Bis-EMA (1.79%)^21^. Those authors explained their results based on the physical characteristics of the polymers. Even though TEGDMA creates a dense polymer network, the network is not homogeneous. As a result, some degree of spatial heterogeneity is expected (some parts of the network show great amounts of crosslinked chains, while others do not, with the formation of microgel domains with highly entangled chains dispersed into unreacted monomers). This phenomenon occurs due to the high speed of polymerization, which leads to rapid formation of a rigid matrix with a large number of pores inside^12^ ^,^ ^26^. In this sense, pre-heating is advantageous because it increases the degree of conversion without accelerating the time at which maximum polymerization occurs, and thus creates a more crosslinked polymer^6^.

The composites here studied present different monomer combinations. Durafill VS, for instance, has only UDMA monomer in its composition, whereas Z-250 and Z-350 contain a combination of UDMA/Bis-GMA/Bis-EMA/TEGDMA, resulting in different fluid absorption behaviors (Table 1). Chains that comprise homopolymers tend to behave differently than those comprising combinations of monomers. Bis-GMA and TEGDMA combined generate a higher degree of conversion when compared with homopolymers consisting only of either monomer^26^. Furthermore, it has been reported that the main path of degradation occurs at the interface between fillers and the resin matrix: filled specimens absorb twice as much water as unfilled specimens^12^. In fact, it has been argued that the interface between the filler and the polymethylmethacrylate resin is the most probable site for the accommodation of additional water. This “grain-boundary” diffusion mechanism leads to hydrolytic degradation of silane^19^, and may affect composite surface properties such as roughness and hardness^4^. The Durafill VS and Z-350 resins contain filler particles with smaller sizes, and therefore show a higher rate of filler/resin interfaces and are more prone to be degraded than Z-250. In addition, the Durafill VS resin contains particles of silica and pre-polymerized silica/resin, again resulting in a greater rate of filler/resin interfaces and probably explaining the more pronounced penetration of silver into these particles. Similarly, Z-350 comprises nanoclusters of silica/zirconia, and therefore presents more degradable interfaces. Figures 5a, 6a, and 7a show the penetration of silver around filler particles, and Figures 5b and 7b, inside pre-polymerized particles, of Durafill VS and Z-350 nanoclusters, respectively.

Other factors that may have influenced the performance of materials in the present study include filler distribution into the resin matrix, optical properties of the fillers, and the amount/quality of the photoinitiator system employed^13^ ^,^ ^14^. Most materials include camphorquinone as a curing initiator, but manufacturers do not report the amount of initiator used or the type and amount of co-initiator employed. In this sense, it would be premature to conclude that the degradation observed for the different assessed composites was determined only by the organic/inorganic matrix ratio, size of filler particles, or type of monomer. Future investigations should be conducted to clarify these aspects.

One possible limitation of this investigation was the absence of a control group (not subjected to degradation). Nevertheless, our findings suggest that degradation really occurred, as the radiopacity of pre-heated composites and those cured at room temperature was similar at baseline, but different after NaOH storage. Further investigations on the same topic should include a control group in an attempt to better understand the extent of degradation and how pre-heating mitigated it.

Some biological concerns exist regarding the application of pre-heated composites *in vivo*. It could be speculated that the temperature of 60°C used to pre-heat composites may be too high for the pulp – it should be noted that a 5.5°C elevation in temperature is capable of damaging tissues irreversibly^30^. However, it has been shown that the use of a pre-heated composite increases intrapulpal temperature in extracted teeth by only 0.8°C considering 1-mm thick dentin discs^7^, and by 4-5°C in 0.5-mm thick dentin discs^9^. In addition, the temperature of the pulpal floor *in vivo* has been shown to increase by 6°C in association with pre-heated composites vs. materials handled at room temperature^24^. The low temperatures reported in those studies probably result from loss of heat during the time elapsed between composite heating and its insertion into the cavity preparation. Although those previous studies suggest that pre-heated composites are suitable for application *in vivo*, important aspects, such as cavity depth, pulp condition, and patient age, among others, have not been investigated. Therefore, more studies are needed before pre-heated composites can be safely used in patients.

## CONCLUSION

The null hypothesis of the present work was rejected: composites cured at 60°C (pre-heated) showed less degradation than those cured at room temperature, regardless of the type of composite used. Degradation occurred in association with both temperatures, but it was mitigated by pre-heating. The alkaline medium proved to be suitable for the evaluation of composite degradation.
